# Supplementary material for: A Unique, Porous C3N4 Nanotube for Electrochemiluminescence with High Emission Intensity and Long-Term Stability: The Role of Calcination Atmosphere
Source: Molecules. 2022 Oct 13;27(20):6863. doi: 10.3390/molecules27206863 (PMC9607187; doi:10.3390/molecules27206863)
Supplement: Supplementary file 1 [file molecules-27-06863-s001.zip › molecules-1958750-supplementary.pdf]

Supplementary materials

A Unique, Porous C_3N_4 Nanotube for Electrochemiluminescence with High Emission Intensity and Long-term Stability: The Role of Calcination Atmosphere

Bolin Zhao, Xingzi Zou, Jiahui Liang, Yelin Luo, Xianxi Liang, Yuwei Zhang * and Li Niu *

Guangzhou Key Laboratory of Sensing Materials & Devices, Center for Advanced Analytical Science, School of Chemistry and Chemical Engineering, Guangzhou University, Guangzhou 510006, China
* Correspondence: ccywzhang@gzhu.edu.cn (Y.Z.); lniu@gzhu.edu.cn (L.N.)

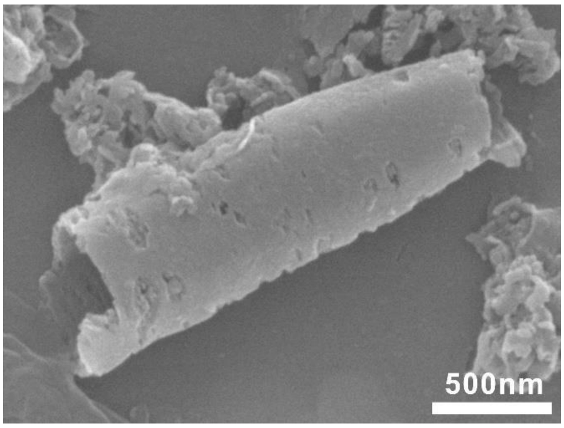

Figure S1. The SEM image of the C_3N_4 nanotube prepared at Air atmosphere.

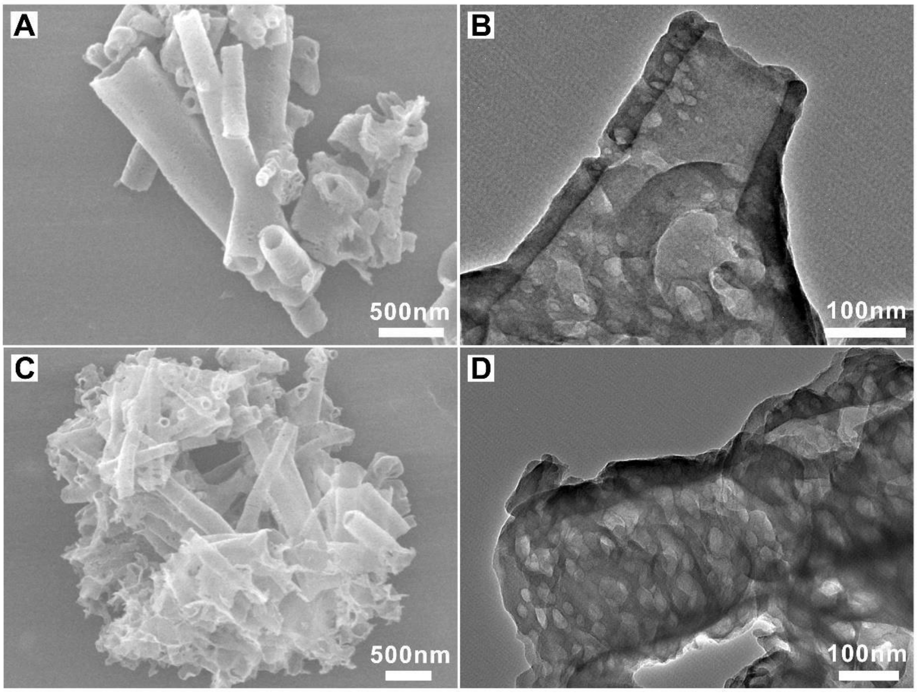

Figure S2. (**A**, **B**) The SEM and TEM images of C_3N_4 nanotube calcination at Ar atmosphere. (**C**, **D**) The SEM and TEM images of C_3N_4 nanotube calcination at N_2 atmosphere.

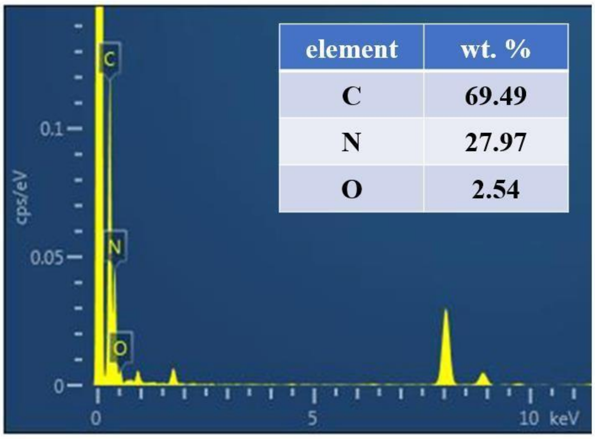

Figure S3. The element content from the EDS spectrum.

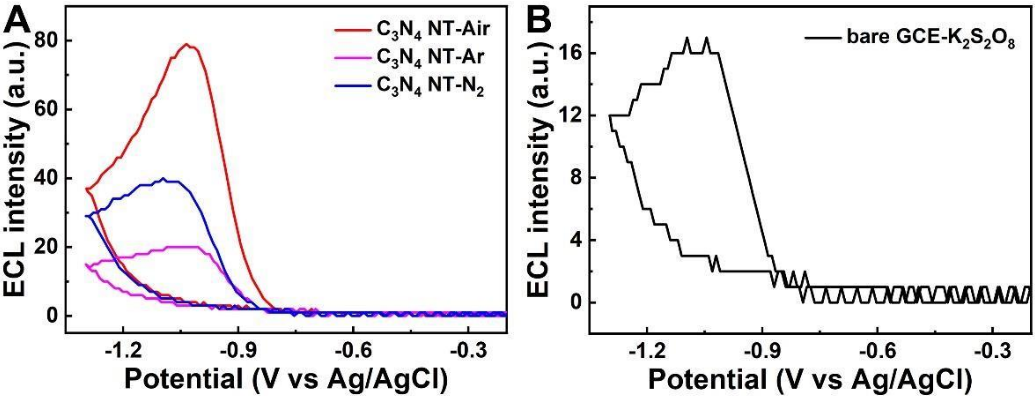

Figure S4. (**A**) ECL response of C_3N_4 NT-Air, C_3N_4 NT-Ar and C_3N_4 NT-N2 modified GCE in 0.1M PBS without $K_2S_2O_8$. (**B**) ECL response of the bare GCE in 0.1M PBS with 100 mM $K_2S_2O_8$.

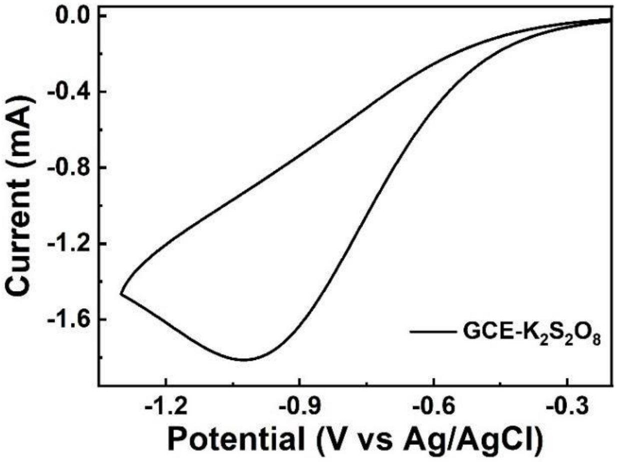

Figure S5. CV curve of the bare GCE in 0.1M PBS with 100 mM $K_2S_2O_8$.

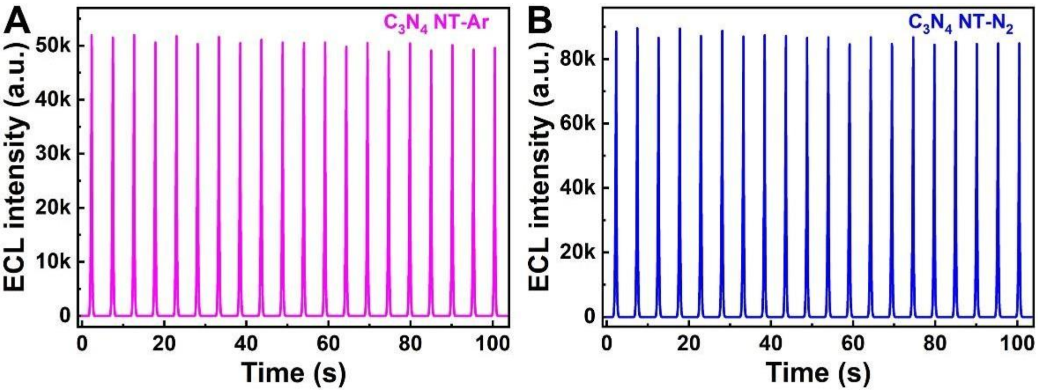

Figure S6. Stability of the C_3N_4 NT-Ar and C_3N_4 NT-N2 modified GCE ECL system for 20 scans.

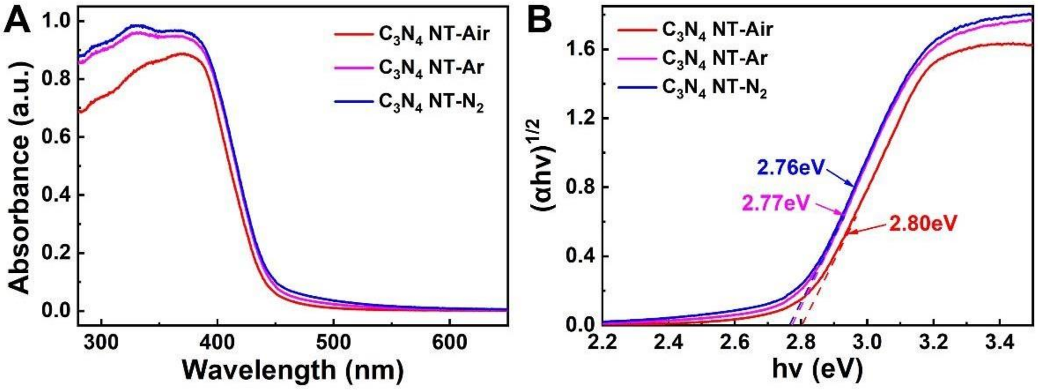

Figure S7. (A) UV-vis DRS and (B) Kubelka-Munk plots for the band gap calculation energies of C_3N_4 nanotube prepared in different atmosphere.

Table S1. Comparison of C_3N_4 NT-Air/$K_2S_2O_8$ ECL sensing system with other ECL system for the detection of Cu^{2+} [1–9].

ECL system	Linear range	LOD	Ref
C_3N_4 NT-Air/$K_2S_2O_8$	0.25~1000 nM	0.08 nM	This work
g-C_3N_4/GQDs	0.5~1000 nM	0.37nM	1
CdTe/CdS QDs	0.1~10 μM	20 nM	2
C,N-QDs@NSs	5×10^{-4}~10 μM	200 nM	3
N-CQDs	0.01~1000μM	0.12μM	4
P-CQDs	0~1000 nM	0.27 nM	5
Au-CNNS	5~500 nM	5 nM	6
CN/1,8-NDI_X	5~10000 nM	0.86 nM	7
CdS/ZnS QDs	2.5~200 nM	0.95 nM	8
BPQDs/$K_2S_2O_8$	0.5~1000 nM	0.07nM	9

References

1. Liu, Y.; Sun, Y.; Yang, M. A double-potential ratiometric electrochemiluminescence platform based on g-C_3N_4 nanosheets (g-C_3N_4 NSs) and graphene quantum dots for Cu^{2+} detection. *Anal. Methods* **2021**, *13*, 903–909.
2. Wang, J.; Jiang, X. Anodic near-infrared electrochemiluminescence from CdTe/CdS coresmall/shellthick quantum dots and their sensing ability of Cu^{2+}. *Sens. Actuators B: Chem.* **2015**, *207*, 552–555.
3. Fang, L.; Xue, Y.; Hu, X.M.; Xie, D.; Li, W.J. Enhanced electrochemiluminescence behavior of C,N quantum dots embedded g-C_3N_4 nanosheets and its sensing application for copper (II). *J. Mater. Sci. Mater. Electron.* **2018**, *29*, 20580–20587.
4. Li, R.; Zhu, Z.; Pan, P.; Liu, J.; Zhou, B.; Liu, C.; Yang, Z.; Wang, J.; Li, X.; Yang, X.; Chang, J.; Niu, H. One-step synthesis of nitrogen-doped carbon quantum dots for paper-based electrochemiluminescence detection of Cu^{2+} ions. *Microchemi. J.* **2022**, *174*, 107057.

5. Venkateswara Raju, C.; Kalaiyarasan, G.; Paramasivam, S.; Joseph, J.; Senthil Kumar, S. Phosphorous doped carbon quantum dots as an efficient solid state electrochemiluminescence platform for highly sensitive turn-on detection of Cu^{2+} ions. *Electrochimi. Acta* **2020**, *331*, 135391.
6. Feng, Y.; Wu, H.; Wei, J.; Chen, L.; Chi, Y. Detection of divalent copper with improved accuracy by dual suppression of electrochemiluminescent recovery. *J. Electro. Chem.* **2019**, *834*, 145–149.
7. Liu, F.Y.; Zhang, T.K.; Zhao, Y.L.; Ning, H.X.; Li, F.S. Electrochemiluminescence of 1,8-Naphthalimide-modified carbon nitride for Cu^{2+} detection. *J. Anal. Test.* **2021**, *6*, 296–307.
8. Zhao, G.; Li, X.; Zhao, Y.; Li, Y.; Cao, W.; Wei, Q. Electrochemiluminescence assay of Cu2+ by using one-step electrodeposition synthesized CdS/ZnS quantum dots. *Analyst* **2017**, *142*, 3272–3277.
9. Chen, J.; Wang, Q.; Liu, X.; Chen, X.; Wang, L.; Yang, W. Black phosphorus quantum dots as novel electrogenerated chemiluminescence emitters for the detection of Cu^{2+}. *Chem. Commun.* **2020**, *56*, 4680–4683.
